# A patient with plasmablastic lymphoma achieving long-term complete remission after thalidomide-dexamethasone induction and double autologous stem cell transplantation: a case report

**DOI:** 10.1186/s12885-018-4561-9

**Published:** 2018-06-08

**Authors:** Alessandro Broccoli, Laura Nanni, Vittorio Stefoni, Claudio Agostinelli, Lisa Argnani, Michele Cavo, Pier Luigi Zinzani

**Affiliations:** Institute of Haematology “L. e A. Seràgnoli”, University of Bologna, Via Massarenti, 9 –40138 Bologna, Italy

## Abstract

**Background:**

No standard of care is established for plasmablastic lymphoma (PBL) and prognosis remains extremely poor, given that patients relapse early after chemotherapy and display resistance to commonly applied cytostatic drugs.

**Case presentation:**

We report a case of nodal, HIV-unrelated PBL in a patient who achieved and maintained a very long lasting complete remission after an intensive therapy consisting consisting of thalidomide plus dexamethasone followed by a consolidation with double autologous stem cell transplantation. Our approach was based on the full application of a standard multiple myeloma treatment and, to the best of our knowledge, it represents the only reported experience so far. This treatment was overall well tolerated.

**Conclusions:**

Multiple myeloma-like treatment may represent a possible alternative to intensive lymphoma-directed therapies.

## Background

Plasmablastic lymphoma (PBL) is a rare and highly aggressive subtype of diffuse large B-cell lymphoma, characterized by diffuse proliferation of large neoplastic cells which resemble B-cell immunoblasts but have a plasma cell immunophenotype [1]. No standard of care is established for this disease and prognosis remains extremely poor, given that patients relapse early after chemotherapy and display resistance to commonly applied cytostatic drugs. We herein report a case of nodal, HIV-unrelated PBL in a patient who achieved and maintained a very long lasting complete remission after an intensive multiple myeloma (MM)-like treatment.

## Case presentation

**Fig. 1:**
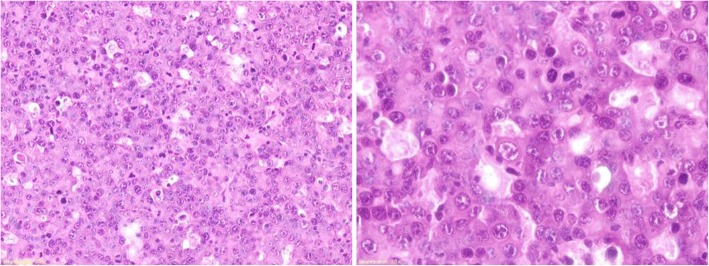
Hematoxylin-eosin staining (20× and 40× magnification) showing macrophages admixed to plasmoblasts conferring a “starry-sky” appearance

**Fig. 2:**
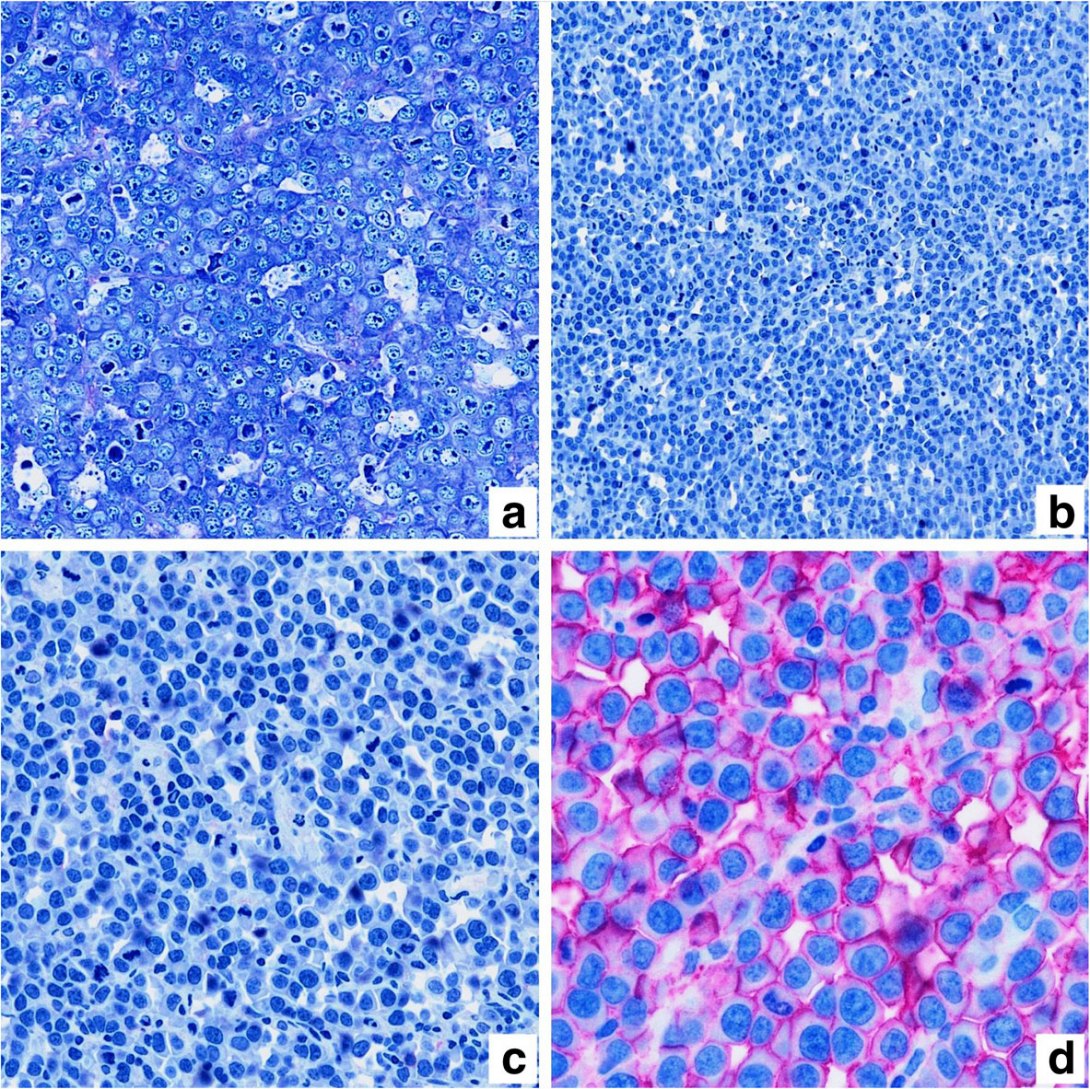
Panel **a**: Giemsa stain (20× magnification). Neoplastic elements are negative for CD20 (panel **b**, 20× magnification) and CD30 (panel **c**, 40× magnification), whereas they stain positively for the plasma cell marker CD138 (panel **d**, 40× magnification)

A 46-year old Italian female presented in June 2007 with 3 enlarged lymph nodes in her left groin without any systemic symptoms. She had no significant comorbidities and no underlying immunosuppression. An excisional biopsy showed diffuse proliferation of large lymphoid cells with several interspersed macrophages, which imparted a “starry-sky” appearance to the infiltrate (Fig. 1). Immunohistochemical examination revealed that tumor cells were negative for CD20, CD79a, CD5, CD30, PAX5, Bcl-2 and Bcl-6, while showing positivity for CD138, MUM1 and λ light chain restriction (Fig. 2). Anaplastic large-cell lymphoma kinase (ALK) and human herpes virus 8 (HHV-8) were also tested by immunohistochemistry, and were both negative. The Ki-67 proliferation index was 98%. Epstein-Barr virus (EBV)-encoded RNA in situ hybridization (EBER) was negative. A diagnosis of PBL was made. A total body computed tomography (CT) scan showed at least five adenopathies in her left groin, the largest of which was 22 × 17 mm. A positron emission tomography (PET) scan confirmed several hypermetabolic lymph nodes in both groins and pelvis (SUVmax 11–14). Biopsy of the iliac crest showed no marrow involvement.

On admission, laboratory data were the following: white blood cells 9500/μL, hemoglobin 12.5 g/dL, platelets 313,000/μL; lactate dehydrogenase, calcium, serum proteins and immunoglobulin levels were all within normal ranges; protein electrophoresis and immunofixation showed no monoclonal component. Serology for human immunodeficiency virus (HIV) was negative.

From July to September 2007, the patient received 3 cycles of thalidomide and dexamethasone: thalidomide was given at the dose of 100 mg/day and dexamethasone at the dose of 40 mg/day on days 1–4 and 9–12 of each cycle. The rationale for the use of thalidomide in PBL relied upon the notion that increased angiogenesis plays a major role in aggressive lymphomas, as well as in MM [2]. The post-induction PET scan showed no metabolically active lesions, thus being compatible with a complete response (CR).

Afterwards, the patient received maintenance therapy with thalidomide for 2 months, followed by peripheral blood stem cell (PBSC) mobilization achieved by the administration of high dose cyclophosphamide (7 g/m^2^) and granulocyte-colony stimulating factor (G-CSF): 7.2 × 10^6^ CD34^+^/kg were harvested after a single apheresis. Following this procedure, the patient went back on thalidomide-dexamethasone for 3 more months, then she underwent an autologous stem cell transplantation (ASCT), conditioned with melphalan, 200 mg/m^2^. 2.5 × 10^6^ CD34^+^/kg PBSC were reinfused. The PET scan performed one month after ASCT confirmed a CR. In light of the good response achieved so far, it was decided to proceed with a second ASCT. The patient again received thalidomide-dexamethasone for 3 months, then 2.5 × 10^6^ CD34^+^/kg PBSC were reinfused 2 days after melphalan conditioning. A CR was clinically and metabolically confirmed by PET scan after the second ASCT. The patient is now healthy and in durable CR after at least 10 years.

This retrospective study was approved by our institutional board and by our Ethical Committee (Comitato Etico Indipendente Policlinico S.Orsola-Malpighi di Bologna) and has been performed in accordance with the ethical standards of the Declaration of Helsinki. Patient provided a written informed consent to publish her data.

## Discussion and conclusions

PBL is a highly aggressive lymphoma strongly associated with immunosuppression, in particular with HIV infection [1]. Nevertheless, PBL has also been described in HIV-negative individuals, usually in association with other causes of immunosuppression (steroid therapy for autoimmune disorders, solid organ transplantation), pre-existing lymphoproliferative diseases or in elderly patients. PBL mainly occurs in men (75%) with a mean age of 39 years in HIV-positive and 58 years in HIV-negative patients [3]. The disease shows a marked propensity to involve the oral cavity, while the most common extraoral sites are the gastrointestinal tract, lymph nodes and skin. The frequency of oral involvement is higher in HIV-positive (58%) than in HIV-negative patients (16%). Other less common localizations include the central nervous system, paranasal sinus, mediastinum, lungs, liver, testes, retroperitoneum, parotid gland and soft tissues. Bone marrow involvement has been reported in 30% of cases in both HIV-positive and negative individuals. Irrespective of the HIV status, approximately 60% of patients present with rapidly progressive, destructive, advanced-stage disease; B symptoms tend to be more common in HIV-unrelated cases [3].

The pathogenesis of PBL is currently unclear. The neoplastic cell derives from a post-germinal centre activated B-cell that has already undergone somatic hypermutation and class switching recombination and is in the process of becoming a plasma cell [1]. Mutations and chromosomal aberrations that lead to the development of the malignancy are likely to occur during this transition. Rearrangements of the oncogene *MYC* occur in up to 47% of HIV-related PBL cases and end up with overcoming the regulatory effects of its repressors Bcl-6 and BLIMP-1, thus allowing cells to proliferate without control. Moreover, *MYC* translocations are thought to contribute to the plasmablastic morphology and to a worse prognosis [4, 5]. The HIV infection seems to correlate with depth and duration of immunodeficiency and the loss of immune control over oncogenic viral infections – specifically EBV and HHV-8. A chronic antigenic stimulation and the presence of a persistent inflammatory state leads in turn to B-cell dysfunctional proliferation and gives rise to a monoclonal population [1]. The strong association with EBV infection is demonstrated by the expression of EBER, detectable in 80 and 46% of HIV-positive and negative cases, respectively.

Making a diagnosis of PBL is always challenging: the immunophenotype resembles that of plasma cell neoplasms, as PBL cells typically lack expression of B-cell markers (CD19, CD20, PAX-5) and show little to no expression of leukocyte common antigen CD45, whereas the plasma cell markers CD79a, IRF4/MUM-1, BLIMP-1, CD38 and CD138 are usually highly expressed. The proliferation rate is generally high, with Ki-67 expression higher than 60% [3]. Association with HIV and/or EBV infection, alongside the absence of monoclonal paraproteinemia, hypercalcemia, renal dysfunction and lytic bone lesions are the key features that favour the diagnosis of PBL instead of MM.

Given its rarity and peculiar features, no standard of care can be established for PBL patients, whose prognosis remains very poor. Early relapses and subsequent chemotherapy resistance characterize the clinical course of the disease. A large literature review of 248 cases of PBL, 50% of which had received cyclophosphamide, doxorubicin, vincristine, prednisone (CHOP) or CHOP-like regimens, while 23% had been treated with more intensive regimens, such etoposide, vincristine, doxorubicin, cyclophosphamide, prednisone (EPOCH), hyperfractionated cyclophosphamide, vincristine, doxorubicin, dexamethasone (hyper-CVAD) and cyclophosphamide, vincristine, doxorubicin, methotrexate alternating with ifosfamide, etoposide, cytarabine (CODOX-M/IVAC), showed an overall response rate to chemotherapy of 77%, with 46% of patients achieving a CR and 31% a partial response [6]. The median progression-free and overall survival (OS) of PBL patients range between 6 and 7 months and 11–13 months respectively, with no differences between CHOP/CHOP-like and more intensive regimens. ASCT may play a role in improving the outcomes of CR patients [7]. Untreated patients invariably die within a few months (median survival: 3 months). In HIV-infected patients, the use of highly active antiretroviral therapy is recommended, as it seems to be associated with improved survival. However, the strongest prognostic factor for these patients remains the achievement of a CR with chemotherapy, which is associated with a median OS of 48 months compared with 3 months for patients without a CR [6].

On the basis of the plasmacytic differentiation of this lymphoma, given that a plasmablast is an activated B-cell undergoing the process of becoming a plasma cell, PBL is ontogenetically collocated between an aggressive post-germinal centre B-cell non-Hodgkin lymphoma and MM. For this reason, the use of antimyeloma agents – like bortezomib, thalidomide or lenalidomide - may have a sound rationale, although the experience with these agents is rather limited. More specifically, they have been predominantly combined with more standard lymphoma-directed regimens, such as CHOP or EPOCH [8–15], rather than applied in MM-like treatment algorithms, also considering at least one ASCT as part of the frontline approach. Case reports accounting for the use of either bortezomib or lenalidomide, applied as single agents or within combinations in both untreated and relapsing PBL patients, have been published in the last few years, sometimes with considerable benefit (Table 1). Our approach was instead based on the full application of a standard MM treatment, with a double ASCT consolidation, and to the best of our knowledge it represents the only reported experience so far. It is important to note, however, that despite the intrinsic aggressiveness of the disease, the early stage at presentation (which accounted for a low International Prognostic Index) may have had a favourable impact on the long-term prognosis of the patient. This treatment was overall well tolerated in an otherwise healthy patient who was considered eligible for a double ASCT, which still represents the standard of care at most Italian institutions for newly diagnosed MM patients who are candidates for an intensive approach. We believe that this approach may represent a possible alternative to intensive lymphoma-directed therapies.

**Table 1:** Recent case reports in which antimyeloma drugs have been applied in HIV-negative PBL patients

Agent	Author, year	Combination	Patients	Setting	Outcomes
Bortezomib	Saba, 2013 [8]	Single agent	1	Salvage	PR; DOR 5 months; death
	Yan, 2014 [9]	Bor + Rituximab + Dex	1	Salvage	nCR; 3+ months post-ASCT
	Castillo, 2015 [10]	Bor + DA-EPOCH	3 (*)	First-line	3 CR; DOR 12+, 18+, 24+ months
	Hirosawa, 2015 [11]	Single agent	1	Salvage	Transient regression
	Fedele, 2016 [12]	Bor + DA-EPOCH	1	First-line	CR; DOR 24+ months
	Cencini, 2016 [13]	Bor + COMP	1	First-line	CR; DOR 12 months
Lenalidomide	Carras, 2015 [14]	Single agent	1	Salvage	CR; DOR 6 months
	Schmit, 2017 [15]	Len + cyclo + dex	1	First-line	CR; DOR 24+ months

(*) 2 HIV-positive patients included

*Bor* bortezomib, *Dex* dexamethasone, *DA-EPOCH* dose-adjusted EPOCH, *COMP* same as CHOP, but with liposomal doxorubicin, *Len* lenalidomide, *Cyclo* cyclophosphamide. *PR* partial response, *(n)CR* (near) complete response, *DOR* duration of response, *ASCT* autologous transplantation. The sign “+” indicates an ongoing response at the moment of the report

## Abbreviations

ASCT: Autologous stem cell transplantation
CHOP: Cyclophosphamide, doxorubicin, vincristine, prednisone
CODOX-M/IVAC: Cyclophosphamide, vincristine, doxorubicin, methotrexate alternating with ifosfamide, etoposide, cytarabine
CR: Complete response
CT: Computed tomography
EPOCH: Etoposide, vincristine, doxorubicin, cyclophosphamide, prednisone
hyper-CVAD: Hyperfractionated cyclophosphamide, vincristine, doxorubicin, dexamethasone
MM: Multiple myeloma
OS: Overall survival
PBL: Plasmablastic lymphoma
PBSC: Peripheral blood stem cell
PET: Positron emission tomography
